# (10*Z*)-Debromohymenialdisine from Marine Sponge *Stylissa* sp. Regulates Intestinal Inflammatory Responses in Co-Culture Model of Epithelial Caco-2 Cells and THP-1 Macrophage Cells

**DOI:** 10.3390/molecules24183394

**Published:** 2019-09-18

**Authors:** Seon Min Lee, Na-Hyun Kim, Sangbum Lee, Yun Na Kim, Jeong-Doo Heo, Jung-Rae Rho, Eun Ju Jeong

**Affiliations:** 1 Gyeongnam Department of Environment & Toxicology, Korea Institute of Toxicology, 17 Jegok-gil, Munsan-eup 52834, Korea; smlee84@kitox.re.kr (S.M.L.); nhkim@kitox.re.kr (N.-H.K.); jdher@kitox.re.kr (J.-D.H.); 2 Department of Oceanography, Kunsan National University, Kunsan 54150, Korea; sblee08@kunsan.ac.kr; 3 Department of Agronomy and Medicinal Plant Resources, Gyeongnam National University of Science and Technology, Jinju 52725, Korea; skdbssk@hanmail.net

**Keywords:** *Stylissa* sp., (10*Z*)-debromohymenialdisine, inflammation, inflammatory bowel disease, co-culture, Caco-2, THP-1

## Abstract

Crohn’s disease (CD) and ulcerative colitis (UC), collectively referred to as inflammatory bowel disease (IBD), are autoimmune diseases characterized by chronic inflammation within the gastrointestinal tract. Debromohymenialdisine is an active pyrrole alkaloid that is well known to serve as a stable and effective inhibitor of Chk2. In the present study, we attempted to investigate the anti-inflammatory properties of (10*Z*)-debromohymenialdisine (**1**) isolated from marine sponge *Stylissa* species using an intestinal in vitro model with a transwell co-culture system. The treatment with **1** attenuated the production and gene expression of lipopolysaccharide (LPS)-induced Interleukin (IL)-6, IL-1β, prostaglandin E2 (PGE2), and tumor necrosis factor-α in co-cultured THP-1 macrophages at a concentration range of 1–5 μM. The protein expressions of inducible nitric oxide synthase (iNOS) and cyclooxygenase (COX)-2 were down-regulated in response to the inhibition of nuclear factor kappa-light-chain-enhancer of activated B cells (NF-kB) translocation into the nucleus in cells. In addition, we observed that **1** markedly promoted the nuclear translocation of nuclear factor erythroid 2 related factor 2 (Nrf2) and subsequent increase of heme oxygenase-1 (HO-1) expression. These findings suggest the potential use of **1** as a pharmaceutical lead in the treatment of inflammation-related diseases including IBD.

## 1. Introduction

The prevalence of inflammatory bowel disease (IBD) including ulcerative colitis (UC) and Crohn’s disease (CD) has been increasing worldwide [1]. The prolonged inflammation of the gastrointestinal tract in IBD leads to the sometimes irreversible impairment of gastrointestinal structure and functions. Though the major forms of IBD, UC, and CD share many clinical and pathological features, they also have markedly different characteristics in their underlying mechanisms. CD is characterized by abdominal pain, diarrhea, narrowing of the gut lumen, and pathological features include transmural inflammation, granulomas, and fistulae that lead to bowel obstruction [2]. Whereas the clinical features of UC include predominantly left-sided lower abdominal pain, diarrhea, rectal bleeding, and frequent and bloody stool [3].

Numerous studies with clinical patients and the credited animal models have revealed that the disturbance of intestinal homeostasis in IBD results from an inappropriate and continuing mucosal inflammatory response to commensal microflora [3]. During the initiation and progression of IBD, an abnormal intestinal immune response with subsequent inflammation are driven by intestinal flora leading to the disruption of the mucosal epithelial barrier [4]. The disturbance of epithelial integrity is known to trigger IBD development via dysfunctions in signal transduction between the immune system and adjacent microflora [5].

As in vitro models for evaluation of intestinal pharmacological and toxicological effects as well as the permeability of compounds, primary cell- [6] and stem cell-based [7] models have been advanced. However, cell monocultures have a limitation in representing the complex structure of the human intestine. Recently, Kämpfer et al. [8] presented an in vitro model that resembles the intestinal environment with a co-culture of the differentiated Caco-2 epithelial cells and THP-1 macrophages. For mimicking the intestinal environment, Caco-2 cells were cultured on the transwell and THP-1 cells were on the underlying plate, and two cells were overlapped and communicated via the transwell. lipopolysaccharide (LPS) was chosen for inflammation on intestinal epithelium rather than PMA (Phorbol-12-Myristate-13-Acetate) + Ionomycin [9,10]. This cell-line derived co-culture system has the advantages of easy accessibility and handling, and it also provides a more representative human-intestine-like model. Moreover, Caco-2 cells are known to offer characteristics analogous to human intestinal tissue such as the transport and permeability of substances [11,12]. 

Marine organisms are increasingly recognized as a new source of pharmacological leads because they produce natural products with a variety of structural and biological diversity [13,14]. Recently, during the search for anti-inflammatory substances from marine natural products including sponge, algae, and microorganisms, the methanol extract of the sponge *Phakellia* sp. showed significant inhibition on nitric oxide (NO) production in co-culture system of Caco-2 and THP-1 cells. By bioactivity-guided fractionation, a pyrrole alkaloid, (10*Z*)-debromohymenialdisine (**1**), was successfully isolated from the butanol soluble fraction using size exclusion chromatography (Sephadex LH-20) and reversed phase column chromatography. Several studies have reported a beneficial potential of **1**: a checkpoint kinase 2 (Chk2) inhibitor that arrests cell cycles involved in DNA repair or apoptosis in cancerous cells [15,16]; the inhibition of human immunodeficiency virus 1 (HIV-1) [17]; antifouling activity against the green mussel *Perna viridis* and the bryozoan *Bugula neritina* [18]; the inhibition against the kinase targets of neurodegenerative disease, CDK5/p25, CK2d, and GSK3b; and potent antibacterial activity [19]. To our knowledge, the anti-inflammatory potential of debromhymenialdosine has not yet been reported. In our in vitro system, **1** effectively reduced the production of inflammatory cytokines without cytotoxicity in the concentrations tested. In the present study, the pharmacological potential of **1** in the treatment of IBD has been evaluated using co-cultured human Caco-2 and THP-1 macrophage cells. 

## 2. Experimental

### 2.1. Materials

The LPS (*Escherichia coli* 0127:B8), dimethyl sulfoxide (DMSO), PMA (2-Mercaptoethanol, phorbol 12-myristate 12-acetate), and Monoclonal Anti-β-Actin antibody were purchased from Sigma-Aldrich (St Louis, MO, USA). Phosphate-buffered saline (PBS), RPMI 1640 medium, MEM medium, fetal bovine serum (FBS), penicillin, and streptomycin were purchased from Gibco Life Technologies (Grand Island, NY, USA). The Griess Reagent System was obtained from Promega (Promega, Madison, WI). Enzyme-linked immunosorbent assay (ELISA) kits for TNF-α, Interleukin (IL)-6, and prostaglandin E2 (PGE2) were purchased from R&D Systems (Minneapolis, MN, USA). IL-1β, TNF-α, IL-6, and GAPDH primers were purchased from Bioneer (Daejeon, Korea). Tin protoporphyrin IX (SnPP) was purchased from Porphyrin Products (Logan, UT, USA). Primary antibodies, including inducible nitric oxide synthase (iNOS), cyclooxygenase (COX)-2, p65, Lamin B, phospho-p65, IκBα, phospho-ERK, ERK, phospho-p38, p38, phospho-JNK, JNK, HO-1, and nuclear factor erythroid 2 related factor 2 (Nrf2) were purchased from Cell Signaling Technology, Inc. (Beverly, MA, USA). The secondary antibody was purchased from Jackson ImmunoResearch Laboratories, Inc (West Grove, PA, USA).

### 2.2. Isolation of (10Z)-Debromohymenialdisine from the Sponge Stylissa sp.

A specimen of the sponge *Stylissa* sp. was collected in Cebu, Philippines, in August, 2006. The research samples were identified by Dr. Ji-Hyun Kim and a voucher (06KNU-PIL-8) was deposited in the Marine Biodiversity Institute of Korea (Seocheon, Korea). The freeze-dried specimen (1.3 kg) was extracted with MeOH (2 L) twice at room temperature and partitioned between double distilled water (DDW) and dichloromethane. The aqueous layer was repartitioned into DDW and butanol. The butanolic layer (22.8 g) was subjected to a reversed phase chromatography with stepwise elution from 50% H_2_O to 100% MeOH to produce the six fractions (I–VI). Fraction I (200 mg), showing the activity, was chromatographed on Sepadex LH-20 to yield seven subfractions (I1–I7), Following this separation, the active subfraction (I5, 30 mg) was separated by reversed phase HPLC to obtain (10*Z*)-debromohymenialdisine (**1**, 4.9 mg) at a retention time of 18 min. The HPLC separation was conducted under the conditions of a Phenomenex C8 (250 × 10 mm) column: gradient elution with solvent from 20% MeOH + 80% H_2_O with 0.1% formic acid to 100% MeOH for 45 min and a flow rate of 1.5 mL/min.

Compound **1**: ^1^H NMR (500MHz, DMSO-*d*_6_) 3.27 (2H, m, H-9), 3.30 (2H, m, H-8), 6.57 (1H, brs, H-3), 7.13 (1H, t, *J* = 2.7 Hz, H-2), 8.09 (1H, t, *J* = 3.9 Hz, H-7), 8.56 (1H, br s, H-15), 9.28 (1H, br s, H-15), 10.88 (1H, br s, H-13), 12.16 (1H, s, H-1); ^13^C NMR (125MHz, DMSO-*d*_6_) 31.5 (C-9), 39.2 (C-8), 109.5 (C-3), 119.7 (C-11), 120.1 (C-4), 122.9 (C-2), 126.8 (C-5), 130.2 (C-10), 154.2 (C-14), 162.9 (C-6), 163.3 (C-12).

### 2.3. Cell Cultures

The human epithelial cell line (Caco-2) was obtained from the American Type Culture Collection (ATCC, Manassas, VA, USA). The cells were maintained in MEM (Gibco BRL, Grand Island, NY, USA) containing 20% (*v*/*v*) fetal bovine serum (FBS) and 1% (*v*/*v*) antibiotics (100 U/mL penicillin and 100 μg/mL streptomycin) at 37 °C with a 5% CO_2_ atmosphere. The human monocytic cell line (THP-1) was purchased from the American Type Culture Collection (ATCC, Manassas, VA, USA). The cell line was cultured in RPMI 1640 (Gibco BRL, Grand Island, NY, USA) containing 10% (*v*/*v*) fetal bovine serum (FBS), 1% (*v*/*v*) antibiotics (100 U/mL penicillin and 100 μg/mL streptomycin), and 0.05 mM 2-Mercaptoethanol in a 5% CO_2_ atmosphere.

### 2.4. Differentiation Assay

To differentiate THP-1 monocytic cells to macrophages, THP-1 cells were treated with 50 ng/mL PMA (2-Mercaptoethanol, phorbol 12-myristate 12-acetate) for three days. The cells were then further incubated in fresh medium without PMA for two days. The procedures were carried out according to a previous publication [20]. 

### 2.5. Co-Culture System

Human epithelial Caco-2 cells were seeded at 3.75 × 10^5^ cells/well onto transwell inserts (0.4 μm pore size; Corning CoStar Corp., Cambridge, MA, USA) and maintained for 14–20 days at 37 °C with a 5% CO_2_ atmosphere. Media was freshly changed every three days until the cells were fully differentiated as checked by the transepithelial/transendothelial electrical resistance (TEER) value (>1200 Ω cm^2^). THP-1 cells were seeded in 6-well plates with 8.5 × 10^6^ cells into the bottom plate and the insert with a fully differentiated Caco-2 monolayer was added into the transwell plate pre-loaded with THP-1 cells. To evaluate the anti-inflammatory effect of the compound to be tested, 1 μg/mL LPS was added on the apical side and then the test compound (concentration as indicated) was added to the basolateral side. After 24 h incubation, the production of nitrite and inflammatory mediators were determined from the THP-1 culture medium of the basolateral part. Additional qPCR and western blot assays were performed with THP-1 cells.

### 2.6. TEER Measurements

TEER value measurements were performed to assess monolayer integrity using a volt-ohm meter consisting of a pair of double chopstick-like electrodes (Millicell-ERS, Millipore, MA, USA). One electrode chopstick was placed in the basolateral part and the other in the apical part at a 90° angle. The resistance of each well was measured in triplicate and the mean value was used for all samples. An insert without cells was used as a blank control. The TEER value was calculated using the following formula: TEER (Ω cm^2^) = Resistance − Blank resistance (Ω) × Membrane surface Area (cm^2^).

### 2.7. Cell Viability Assay

Cell viability was measured using the CCK-8 kit (Cell Counting Kit-8), according to the manufacturer’s instructions. Briefly, the cells were plated in 96-well plates at a density of 1 × 10^4^ cells/well. After overnight incubation, the cells were treated with the compound to be tested (at the concentration as indicated) and cultured for 24 h. After incubation, the CCK-8 solution was added to each well and incubated for 3 h at 37 °C. The absorbance at 450 nm was measured using a microplate reader (Bio-Tek Company, Winooski, VT, USA). The experiments were performed in triplicate.

### 2.8. NO Production

The nitrate produced in the culture medium was determined by the Griess assay (Promega, Madison, WI, USA). The Griess reagent was prepared by mixing equal volumes of sulfanilamide solution and NED Solution. For the assay, 100 μL of culture supernatant was mixed with 100 μL of 1× Griess reagent and then incubated for 10 min at room temperature under dark. Afterincubation, the optical density was determined at 540 nm with a microplate reader (Bio-Tek Company, Winooski, VT, USA).

### 2.9. Enzyme-Linked Immuno-Sorbent Assay (ELISA)

The concentration of IL-1β, TNF-α, and IL-6 was quantified using culture supernatant from the basolateral for 24 h of co-culture. The levels of IL-1β, TNF-α, and IL-6 were determined by using an ELISA kit (R&D System, Minneapolis, MN, USA). The absorbance was measured using a microreader (Biotek Instruments, Inc., Winooski, VT, USA) at 450 nm.

### 2.10. qRT-PCR

Total RNA was extracted from THP-1 cells using Trizol reagent (Invitrogen, Carlsbad, CA, USA). Reverse transcription of mRNA into cDNA was performed with a QuantiTect Reverse Transcription Kit (Qiagen, Valencia, CA, USA) according to the manufacturer’s instructions. Quantification was performed with Mx3005P QPCR Systems (Agilent Technologies, Santa Clara, CA) using GoTaq qPCR Master Mix (Promega, Mannheim, Germany) with specific primers. qRT-PCR reactions were performed (initial denaturation at 95 °C for 2 min, 40 cycles at 95 °C for 15 s and at 60 °C for 60 s). The data of each gene was normalized to the housekeeping gene Glyceraldehyde 3-phosphate dehydrogenase (GAPDH).

### 2.11. Western Blotting Analysis

Cells were seeded in 6-well plates with 3 × 10^5^ cells and then treated with the compound to be tested (0~10 μg/mL). After 24 h, cells were washed with cold PBS and extracted with RIPA buffer containing a 1× protease inhibitor cocktail (Santa Cruz, CA, USA) for 40 min on ice. Protein lysates were centrifuged at 13,000× *g* for 30 min at 4 °C. Thirty micrograms of the lysed proteins were separated by SDS-PAGE (8–12%) at 100V and transferred to a PVDF membrane. The membrane was blocked with 5% non-fat milk in PBST buffer for 1 h at room temperature. The membrane was incubated with specific primary antibodies at 4 °C overnight. Then, the membrane was washed three times with PBST followed by incubation with secondary antibodies for 1 h at room temperature. Bands were visualized using ECL solution (Thermo Scientific) and quantified using the Chemidoc Imaging System (Bio-Rad; Hercules, CA, USA).

### 2.12. Statistical Analysis

The results were analyzed using Prism version 5.00 software (GraphPad Software, San Diego, CA, USA). One-way ANOVA was applied to calculate the significance between the groups. Statistical significance was indicated by a P value of <0.05. Data were expressed as the mean ± SEM of three independent experiments.

## 3. Results

### 3.1. Anti-inflammatory (10Z)-Debromohymenialdisine from Stylissa sp. Using Bioactivity-Guided Separation

**Figure 1:**
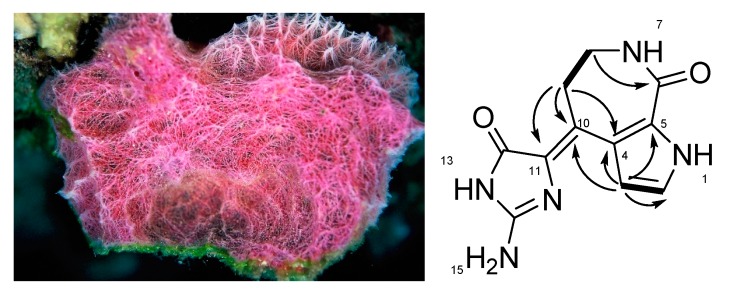
Marine sponge *Stylissa* sp. (left) and the structure of **1** with key COSY (bold lines) and HMBC (arrows) correlations.

Compound **1**, a pale yellow powder, was isolated with phakellistatin and phakellin from the sponge *Stylissa* sp. and was given as the molecular formula of C_11_H_11_N_5_O_2_ on the basis of the protonated peak ([M + H]^+^ = 246.0991, Δ = 0.5) in the positive HRESIMS spectrum. The structure of **1** was determined using HSQC, COSY, and HMBC spectra along with the molecular formula (Figure 1). Proton and carbon chemical shifts were assigned and matched within a small difference by comparison of reported values for (10*Z*)-debromohymenialdisine [21].

**Figure 2:**
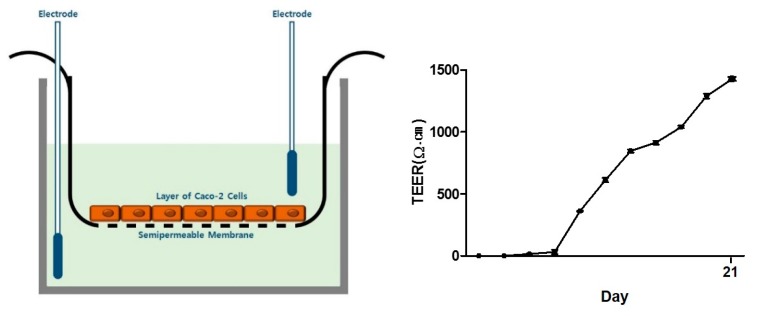
TEER value of endothelial Caco-2 cells. Caco-2 cells were seeded on to transwell inserts and maintained for 14–20 days. The TEER value was measured every two days using a volt-ohm meter with a double chopstick-like electrode. TEER, Transepithelial/transendothelial electrical resistance.

To perform reliable in vitro assays, a co-culture system of endothelial Caco-2 and THP-1 macrophage cells was established. Before the assay, TEER values were measured to first confirm and quantify the barrier integrity of the endothelial monolayer. From seven days after cell plating, the TEER value increased steadily, and full differentiation of Caco-2 cells was found at the 20th day (TEER value > 1200 Ω cm^2^) (Figure 2).

**Figure 3:**
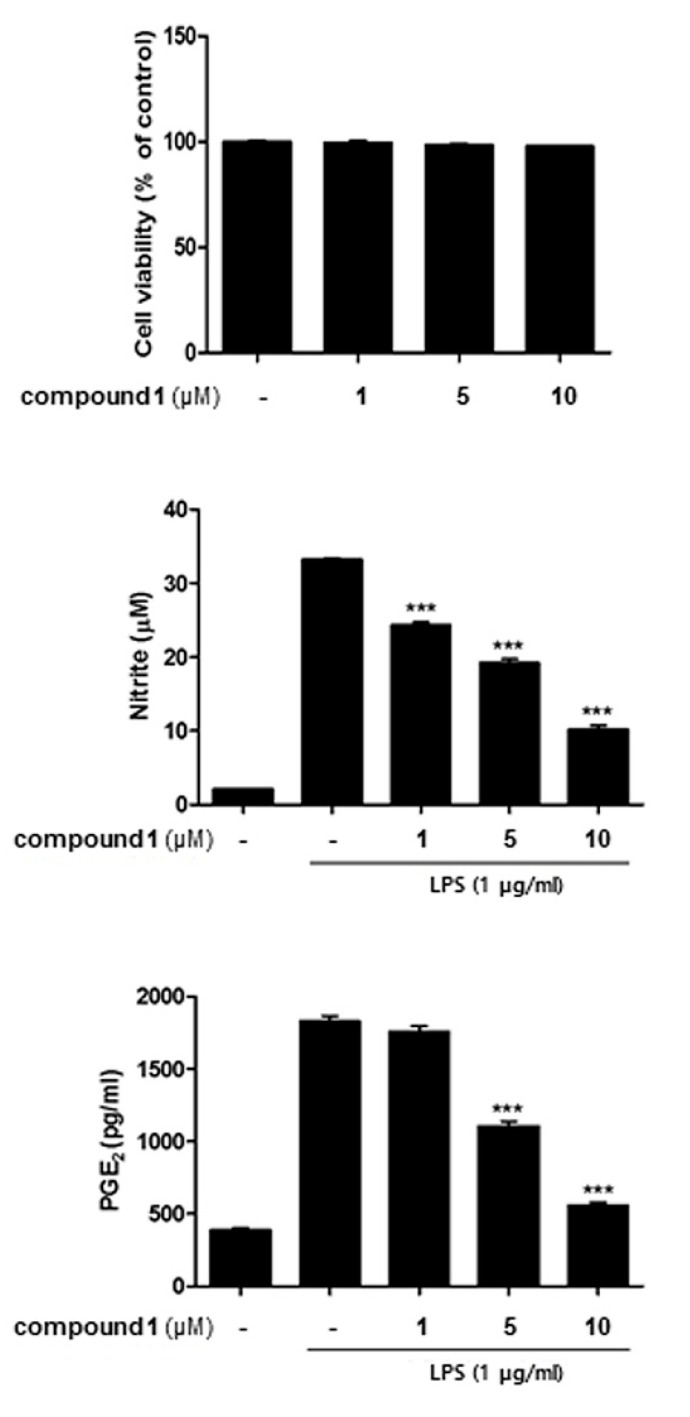
Compound **1** attenuated nitrite production of THP-1 macrophages without cytotoxicity in the co-culture system. Caco-2 and THP-1 cells were co-cultured and various concentrations of **1** (1, 5, and 10 μM), which was treated to the apical compartment of plate. After 1 h, a 100 ng/mL LPS insult was applied to the basolateral compartment, and cells were further incubated overnight. The level of nitrite collected from the culture medium was measured, and then cell viability was measured using CCK-8 staining. *** *p* < 0.001.

Cells were treated with various concentrations of **1** (1, 5, 10 μM) for 1 h, followed by LPS (100 ng/mL) insult. 24 h after LPS treatment, NO levels in the culture medium were increased by twelve-fold and PGE2 levels were increased by four-fold. The pretreatment of cells with **1** led to a significant decrease in the production of LPS-induced NO and PGE2 in a concentration dependent manner (Figure 3). Subsequently, CCK-8 assays were performed, and results indicated that **1** did not show cytotoxicity (cell viability > 98% of non-treated control) in the concentration range tested in the treated cells. These results showed that **1** attenuated the production of NO without cytotoxicity at the concentrations tested (1~10 μM).

### 3.2. (10Z)-Debromohymenialdisine Down-Regulated the Production of Inflammatory Cytokines in a Co-Culture System of Caco-2 and THP-1 Macrophages

**Figure 4:**
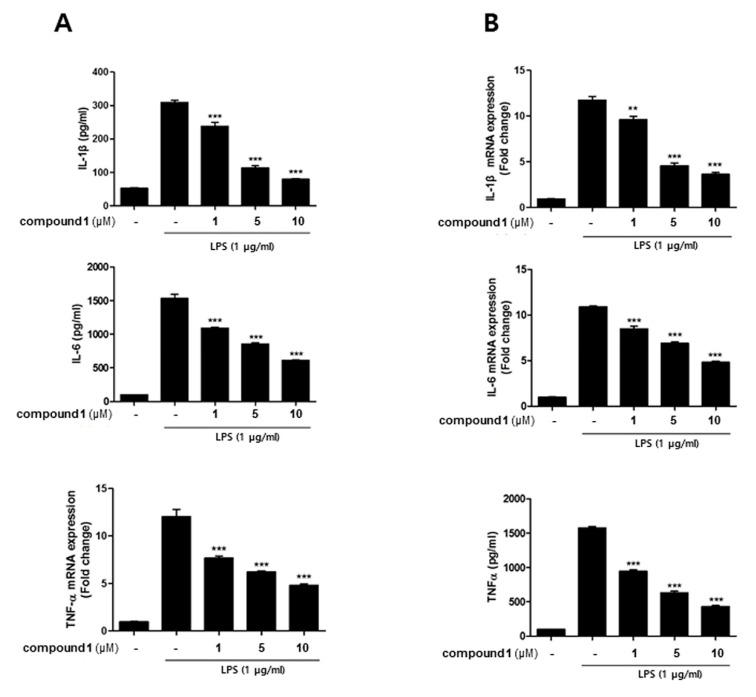
Compound **1** inhibited the production of inflammatory cytokines in THP-1 cells from the co-culture system with Caco-2 cells. THP-1 macrophages were co-cultured with Caco-2 cells in the presence of various concentrations of **1** (1, 5, and 10 μM) that were applied to the apical compartment of the plate. After 1 h of test sample treatment, a 100 ng/mL LPS insult was applied to the basolateral compartment of the plate, and cells were further incubated for 24 h. (**A**) The levels of TNF-α, prostaglandin E2 (PGE2), Interleukin (IL)-1β, and IL-6 in the culture medium were subsequently measured using commercially available ELISA kits. (**B**) The expression of TNF-α, IL-1β, and IL-6 and were analyzed using qRT-PCR. The quantified expression of each mediator normalized to GAPDH is presented (right panel). LPS, lipopolysaccharide; GAPDH, glyceraldehydes-3-phosphate dehydrogenase. ** *p* < 0.01, *** *p* < 0.001.

In a co-culture system of human epithelial Caco-2 cells and THP-1 macrophages, the crosstalk between the two cell types was mediated through soluble messengers secreted into the media. Correspondingly, to further elucidate whether **1** affects inflammatory mediators, the levels of IL-1β, IL-6, and TNF-α were detected following LPS-stimulation. Similar to NO production, the increased level of IL-1β, IL-6, and TNF-α induced by LPS (Figure 4A) decreased in a dose-dependent manners in **1** (1, 5, 10 uM)-treated cells. The effect of **1** on mRNA levels of cytokines was also analyzed using PCR. Pretreatment of cells with **1** resulted in a downregulation of LPS-induced mRNA expressions of IL-1β, IL-6, and TNF-α in concentration-dependent manners (Figure 4B).

### 3.3. (10Z)-Debromohymenialdisine down-Regulated the Expression of iNOS and COX-2 Induced by LPS in Co-Culture System of Caco-2 and THP-1 Macrophages

**Figure 5:**
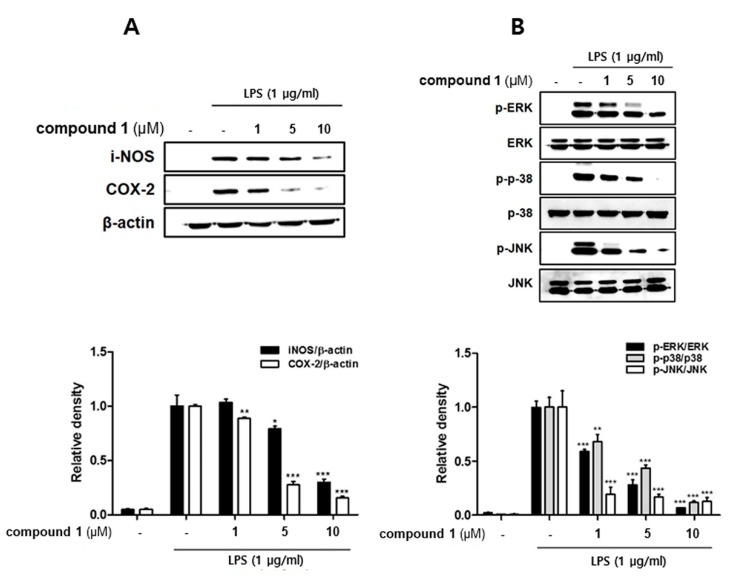
Compound **1** down-regulated the expression of Inducible nitric oxide synthase (iNOS) and cyclooxygenase (COX)-2, and decreased the phosphorylation of mitogen-activated protein kinase (MAPK) in THP-1 cells from the co-culture system with Caco-2 cells. Treatment with **1** (1, 5, and 10 μM) was applied to the apical compartment of the plate. After 1 h, a 100 ng/mL LPS insult was applied to the basolateral compartment, and cells were further incubated for 24 h. (**A**) The protein expression of iNOS and COX-2, phospho-IκBα, and IκBα were then measured by western blot analysis. (**B**) After 15~30 min of LPS insult, the phosphorylation levels of MAPKs (ERK, p38, JNK) were detected by western blot. The representative blot of each mediator is shown (left panel) and the quantified ones (right panel) are shown after normalization to α-tubulin. LPS; lipopolysaccharide. * *p* < 0.05, ** *p* < 0.01, *** *p* < 0.001.

It is well-known that LPS-induced NO overproduction in macrophages is catalyzed by iNOS during the inflammation process [22]. Furthermore, the production of PGE2 is mediated by the induction of COX-2 activity and expression in the activated macrophages [23]. To identify anti-inflammatory properties of **1**, the expression levels of iNOS and COX-2 proteins were detected using western blotting. The insult of LPS up-regulated the expression of pro-inflammatory proteins iNOS and COX-2, whereas pretreatment of cells with **1** (1, 5, 10 uM) significantly attenuated LPS-induced expressions of both inflammatory enzymes iNOS and COX-2 (Figure 5A). We next examined the effect of **1** on the phosphorylation of mitogen-activated protein kinase (MAPK) family, specifically p38, ERK1/2, and JNK. The phosphorylation of p38, ERK1/2, and JNK induced by an LPS insult was significantly attenuated by **1** in a concentration-dependent manner (Figure 5B).

### 3.4. (10Z)-Debromohymenialdisine Inhibited Nuclear Translocation of NF-κB via Increased Interactions with IκB-α

**Figure 6:**
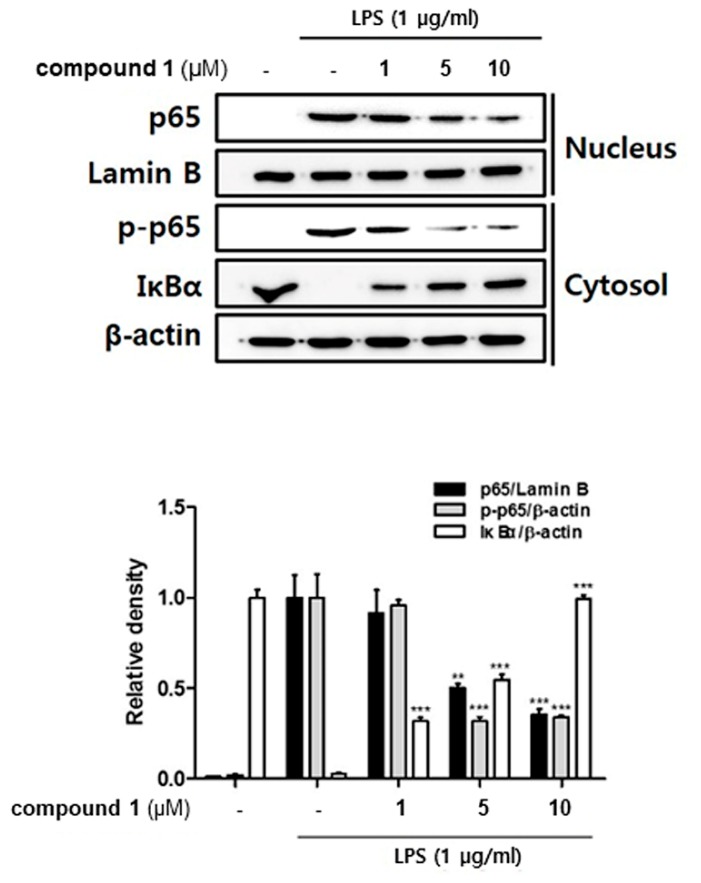
Compound **1** inhibited nuclear factor kappa-light-chain-enhancer of activated B cells (NF-kB) translocation into the nucleus in THP-1 cells from the co-culture system with Caco-2 cells. THP-1 macrophages were co-cultured with Caco-2 cells in the presence of various concentrations of **1** (1, 5, and 10 μM) that were applied to the apical compartment of the plate. After 1 h of test sample treatment, a 100 ng/mL LPS insult was applied to the basolateral compartment of the plate, and the cells were further incubated for 24 h. The cell lysates were separated into nuclear and cytoplasmic fractions, and the expression of p65, phosphorylated-p65, Lamin B, and IκBα were analyzed by western blot. The representative blot is shown (upper panel) and the quantified ones are shown with normalization to α-tubulin (bottom panel). LPS; lipopolysaccharide. ** *p* < 0.01, *** *p* < 0.001.

The NF-κB p65/p50 heterodimer, which contains the transcriptional activation domain to bind DNA, plays a key role in inflammation and immune responses. The activation of NF-κB depends on the phosphorylation-induced degradation of IκB-α, an inhibitor of NF-κB [8,24]. We investigated whether **1** affects the translocation of NF-κB into the cell nuclei and the binding to IκB-α. Followed by treatment with **1** (1, 5, 10 uM), the whole cell lysate was biochemically fractionated into nuclear and cytoplasmic extracts to measure the localization of both p65 and phosphorylated p65. As shown in Figure 6, western blot analysis showed that pretreatment with **1** inhibited the phosphorylation of p65 in the cytoplasmic fraction and attenuated the translocation of p65 into nuclei. Intriguingly, **1** significantly up-regulated total protein levels of IκB-α in thef cytoplasm, which may result in the enhanced binding of IκB-α to p65.

### 3.5. (10Z)-Debromohymenialdisine Increased HO-1 Expression via Nuclear Translocation of Nrf2 In Co-Culture System of Caco-2 and THP-1 Macrophages

**Figure 7:**
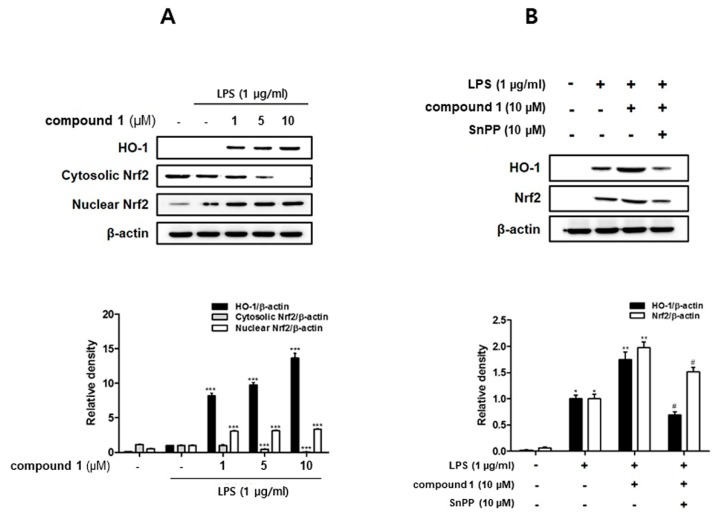
Compound **1** increased HO-1 expression and induced the nuclear translocation of nuclear factor erythroid 2-related factor 2 (Nrf2) in in THP-1 cells from the co-culture system with Caco-2 cells. THP-1 macrophages were co-cultured with Caco-2 cells in the presence of various concentrations of **1** (1, 5, and 10 μM) that were applied to the apical compartment of the plate. After 1 h, 100 ng/mL LPS was added to the basolateral compartment of the plate. (**A**)The expression of HO-1 and Nrf2 were individually analyzed by western blot after 12 h and 1 h of LPS insult, respectively. Compound **1** (10 μM) was added into the apical compartment of the plate in the absence or presence of SnPP, the HO-1 inhibitor. (**B**) The expression of HO-1 and Nrf2 were analyzed by western blot after 12 h and 1 h of LPS insult, respectively. * *p* < 0.05, ** *p* < 0.01, # *p* < 0.05 (compare to the LPS only wells).

HO-1 and its product, carbon monoxide, are known to inhibit the expressions of pro-inflammatory enzymes iNOS and COX-2 [25] and attenuate the production of pro-inflammatory cytokines in the activated macrophages [26]. In order to identify the involvement of (10*Z*)-debromohymenialdisine-associated HO-1 regulation, western blot analysis was conducted. As shown in Figure 7A, following the pretreatment of **1**, the protein levels of HO-1 in THP-1 cells were significantly increased at the concentrations tested (1, 5, 10 uM). The transcription factor nuclear factor erythroid 2-related factor 2 (Nrf-2) is a critical and indispensable regulator of HO-1 [27]. Induction of the Nrf-2/HO-1 pathway has been known to suppress oxidative stress and inflammatory responses in LPS-activated macrophages. Therefore, western blot analysis was performed to determine the effect of **1** on inducing the nuclear translocation of Nrf2. As shown in Figure 7B, the level of Nrf2 in cytoplasmic fraction was sharply decreased, with a concomitant increase in the nuclear fraction. The induction of HO-1 expression and Nrf2 nuclear translocation by **1** treatment was remarkably attenuated by Snpp, a HO-1 inhibitor.

## 4. Discussion

*Stylissa* sp. is a marine sponge that is well known to produce a number of bioactive pyrrole alkaloids including hymenialdisine and debromohymenialdisine [28,29], which are also produced by marine sponge *Axinella* sp. and the genus *Phakellia.* Hymenialdisine, and debromohymenialdisine have been reported to exhibit anti-viral [13] and anti-cancer activity, and serves as a growth stimulator of agricultural plants [30]. More specific to anti-cancer activity, hymenialdisine, debromohymenialdisine, and their derivatives are well known to serve as relatively stable effective inhibitors of Chk2 [13,31,32,33]. The activation of Checkpoint kinase 2 (Chk2) in response to DNA damage leads to either cell cycle arrest, activation of DNA repair, or sometimes apoptosis when severe DNA damage occurs. Several hymenialdisine derivatives with Chk2 inhibitory activity increased the survival of normal cells following exposure to DNA-damaging radiation [14]. A further look into the action mechanisms of debromohymenialdisine in cancer cells including combination therapy with existing medicines and synthesis of potent derivatives have been reported [34,35]. Nevertheless, the bioactivity of debromohymenialdisine is limitedly assigned to anti-cancer activity. In the present study, we attempted to expand the potential utility of (10*Z*)-debromohymenialdisine (**1**) beyond the detection of anti-cancer activity. To our knowledge, this is the first study to identify molecular mechanisms that account for intestinal anti-inflammatory effects of **1**. For evaluation of the anti-inflammatory properties of **1**, an in vitro co-culture system that resembles the intestine was established using human-originated epithelial-like Caco-2 and THP-1 macrophage cells. Then, the effects of **1** on the regulation of NF-κB localization, HO-1 expression, and the related production of pro-inflammatory proteins and cytokines were observed.

Aberrant and continuing inflammatory responses to commensal microflora in a genetically susceptible host is thought to be a major cause of IBD. The abundant evidence supports that ulcerative colitis is a T_H_2-mediated disease, whereas Crohn’s disease is associated with T_H_1-mediated immune response [3]. In IBD patients, the immune cells were found to release abnormally high levels of pro-inflammatory cytokines compared with those of normal tissues [36]. In patients with Crohn’s disease, the markedly increased amounts of IFN-γ together with markedly decreased levels of IL-4 are observed in T cells isolated from the affected tissues [37]. IFN-γ and IL-4, the putative initiating cytokines, affect downstream effector cells including macrophages that are activated to produce pro-inflammatory mediators TNF-α, IL-1β, and IL-6. Furthermore, the markedly enhanced secretion of these downstream pro-inflammatory cytokines is observed in patients with ulcerative colitis and Crohn’s disease [38]. The overexpression of TNF-α and IL-1β has been viewed to play a critical role in IBD pathogenesis [39]. Correspondingly, the agent to block the overproduction of these cytokines is proposed to be effective for IBD treatment. The first choice of drugs for mild-to-moderate IBD patients are anti-inflammatory agents such as aminosalicylates and corticosteroids. Thought aminosalicylates (sulfasalazine or mesalamine) are known to be effective for limiting inflammation in the digestive track, it is limited by its side effects including headache, nausea, and diarrhea in high doses. In the present study, LPS has been used as a stimulator for the Caco-2 and THP-1 co-culture model instead of PMA+ionomycin treatment, as LPS is known to reproduce similar cellular responses in IBD such as the increase of intestinal tight junction permeability and localization of TLR-4 [40]. As a result, it was observed that **1** inhibited the LPS-stimulated production of pro-inflammatory mediators TNF-α, IL-1β, and IL-6 in THP-1-differentiated macrophages co-cultured with Caco-2 cells. Moreover, the expression of corresponding mRNAs was markedly suppressed by **1** in a dose-dependent manner. The increased expression of pro-inflammatory proteins iNOS and COX-2 is involved in the pathogenesis of colitis [41,42]. In the co-culture system, treatment with **1** significantly suppressed the secretion of NO and PGE2 in response to the inhibition of iNOS and COX-2 protein expressions in THP-1 macrophages.

NF-kB is an essential and ubiquitous transcription factor for controlling various genes involved in inflammatory responses [43,44]. The binding sites for NF-kB are located in the proximal region of COX-2 and TNF-α promoters [45,46]. Substances with inhibitory activity on the DNA-binding activity of NF-κB p65 effectively attenuated the expression of iNOS, COX-2, and TNF-α in macrophages [47]. Based on the inhibitory effects of **1** on the expression of pro-inflammatory cytokines and proteins, the regulatory effects of **1** on NF-κB localization in the cell nucleus and cytoplasm has been observed. In an inactivated cell, the NF-κB complex comprising thesubunits p50 and p65 is located in the cytoplasm combined with IκB-α, its inhibitor protein. When cells are activated by inflammatory stimuli, IκB-α is phosphorylated by IkB kinase (IKK) complex and dissociated from NF-κB, allowing the translocation of NF-κB into the nucleus. The translocated NF-κB promotes subsequent transcription and expression of pro-inflammatory mediators [48,49]. Our results demonstrated that **1** disrupts the interactions of NF-kB p65 with a specific set of genes to mediate an inflammatory response in macrophages. In the co-culture system, treatment with **1** inhibited the phosphorylation of p65 and upregulated the protein levels of IκB-α in the cytoplasm, which may induce the interactions of the p65 subunit of NF-kB with IκB-α. The enhanced binding of IκB-α with p65 consequently leads to the cytoplasmic retention of the NF-κB complex, which may prevent the accessibility of NF-κB into the nucleus to bind the target gene promoter. These results suggest that **1** inhibits the expression of pro-inflammatory mediators including iNOS, TNF-α, IL-1β, and IL-6 via regulation of NF-κB pathway.

Recently, the important role of Nrf-2 and the target gene HO-1 as anti-inflammatory effectors has been demonstrated in animal experimental models of IBD. In the dextran sulphate sodium (DSS)-induced colitis mouse model, the enhanced expression of Nrf-2 and HO-1 were detected under colitis wound repair [50]. In the 2,4,6-trinitrobenzenesulfonic acid (TNBS)-induced colitis mouse model, oral administration of lyophilized microalgal biomass from *Chlamydomonas debaryana* up-regulated the expressions of Nrf-2 and HO-1 [51]. In the present study, we observed that **1** markedly promoted the nuclear translocation of Nrf2 and subsequent increase in the expression of HO-1.

## 5. Conclusions

In a co-culture in vitro system resembling the intestinal environment established with epithelial Caco-2 cells and THP-1 macrophage cells, the treatment of **1** exhibited potent anti-inflammatory activity in suppressing LPS-induced expressions of pro-inflammatory (TNF-α, IL-6, IL-1β) and anti-inflammatory (Nrf-2 and HO-1) mediators. Compound **1** down-regulated the expression of iNOS and COX-2, as well as attenuated the nuclear translocation of NF-κB. Considering NF-κB has been viewed as a master regulator in macrophage-related inflammation, these studies support the potential of (10*Z*)-debromohymenialdisine as a therapeutic agent in the treatment of IBD.

## Abbreviations

Chk2	Checkpoint kinase 2
IL	Interleukin
PGE2	Prostaglandin 2
NO	Nitric oxide
iNOS	Inducible nitric oxide synthase
COX	Cyclooxygenase
NF-kB	Nuclear factor kappa-light-chain-enhancer of activated B cells
Nrf2	Nuclear factor erythroid 2 related factor 2
MAPK	Mitogen-activated protein kinase
HRESIMS	High-Resolution ElectroSpray Ionization Mass Spectrometry
HSQC	Heteronuclear Single Quantum Coherence
COSY	COrrelation SpectroscopY
HMBC	Heteronuclear Multiple Bond Correlation
